# Systemic Inflammation, Oxidative Damage to Nucleic Acids, and Metabolic Syndrome in the Pathogenesis of Psoriasis

**DOI:** 10.3390/ijms18112238

**Published:** 2017-10-25

**Authors:** Lenka Borska, Jan Kremlacek, Ctirad Andrys, Jan Krejsek, Kvetoslava Hamakova, Pavel Borsky, Vladimir Palicka, Vit Rehacek, Andrea Malkova, Zdenek Fiala

**Affiliations:** 1 Department of Pathological Physiology, Charles University, Faculty of Medicine in Hradec Kralove, 500 03 Hradec Kralove, Czech Republic; kremlack@lfhk.cuni.cz (J.K.); BORSKYP@lfhk.cuni.cz (P.B.); 2 Department of Clinical Immunology and Allergology, Charles University, Faculty of Medicine in Hradec Kralove, 500 03 Hradec Kralove, Czech Republic; andrys@lfhk.cuni.cz (C.A.); KrejsekJ@lfhk.cuni.cz (J.K.); 3 Clinic of Dermal and Venereal Diseases, Charles University Hospital Hradec Kralove, 500 05 Hradec Kralove, Czech Republic; kveta.hamakova@fnhk.cz; 4 Institute of Clinical Biochemistry and Diagnostics, Charles University Hospital and Faculty of Medicine in Hradec Kralove, 500 05 Hradec Kralove, Czech Republic; vladimir.palicka@fnhk.cz; 5 Department of Transfusion Medicine, Charles University Hospital in Hradec Kralove, 500 05 Hradec Kralove, Czech Republic; vit.rehacek@fnhk.cz; 6 Department of Hygiene and Preventive Medicine, Charles University, Faculty of Medicine in Hradec Kralove, 500 03 Hradec Kralove, Czech Republic; malka8ar@lfhk.cuni.cz (A.M.); fiala@lfhk.cuni.cz (Z.F.)

**Keywords:** psoriasis, inflammation, oxidative stress, metabolic syndrome

## Abstract

In the pathogenesis of psoriasis, systemic inflammation and oxidative stress play mutual roles interrelated with metabolic syndrome (MetS). This study aims to map the selected markers of inflammation (C-reactive protein (CRP)), oxidative damage to nucleic acids (DNA/RNA damage; 8-hydroxy-2′-deoxyguanosine, 8-hydroxyguanosine, and 8-hydroxyguanine), and the parameters of MetS (waist circumference, fasting glucose, triglycerides, high-density lipoprotein (HDL) cholesterol, diastolic and systolic blood pressure) in a group of 37 patients with psoriasis (62% of MetS) and in 43 healthy controls (42% of MetS). Levels of CRP, DNA/RNA damage, fasting glucose, and triglycerides were significantly elevated in patients. MetS in conjunction with psoriasis was associated with high levels of CRP, significantly higher than in control subjects without MetS. Patients with MetS exhibited further DNA/RNA damage, which was significantly higher in comparison with the control group. Our study supports the independent role of psoriasis and MetS in the increase of CRP and DNA/RNA damage. The psoriasis contributes to an increase in the levels of both effects more significantly than MetS. The psoriasis also diminished the relationship between CRP and oxidative damage to nucleic acids existent in controls.

## 1. Introduction

Non-communicable diseases are chronic diseases with a generally slow progression. They are associated with genetic state, lifestyle, and various environmental factors. According to the revised version of World Health Organization (WHO), non-communicable diseases are the leading cause of death and disability worldwide [1]. In 2014, WHO described psoriasis as a serious chronic non-communicable inflammatory skin disease with multifactorial etiology [2]. Recently, the worldwide prevalence of psoriasis in adults ranged from 0.51% to 11.43% [2].

C-reactive protein (CRP) is a key biomarker of acute-phase systemic inflammation and risk for future vascular disease [3]. An elevated level of CRP can be used as a predictor of inflammation in different diseases, including psoriasis [4,5,6,7,8]. It was found that the level of CRP increases with the increasing number of signs of metabolic syndrome (MetS) [9].

Common to non-communicable disease processes is excessive oxidative stress with high levels of reactive oxygen species (ROS) [10]. ROS are reactive endogenous small molecules as by-products of normal aerobic metabolism and the inflammatory/immune response to various challenges that are known to induce damage to cellular components including strand breaks and base modifications in nucleic acids [11,12]. During these processes, it is possible that the signaling processes in the cells are impaired, leading to accelerated cellular aging [13,14]. Oxidative stress (oxidative damage to nuclei acids, DNA/RNA) has been widely investigated and is strongly implicated in the development of many chronic diseases, such as psoriasis [15].

Psoriasis is a multisystem chronic disease connected to different comorbidities like obesity [16]. Obesity is a relevant risk factor for the development and complication onset of non-communicable diseases and their multifactorial etiology has been standing out with high prevalence in all age groups [17]. Obesity is a predicting risk factor for MetS, which is known as a complex disorder combining abdominal obesity, glucose intolerance/resistance (diabetes mellitus), atherogenic dyslipidemia, and hypertension [18]. The existing literature shows that psoriasis is associated with an increased prevalence of MetS [4,19]. It is known that oxidative stress can be considered as an important component of action of MetS [11].

From the facts mentioned above, it is evident that systemic inflammation, oxidative damage to nucleic acids, and MetS are related to the pathogenesis of psoriasis. However, there are unanswered questions about their mutual relationships in the pathogenesis and in the progression of disease and associated complications [11]. Knowledge of these relationships could significantly help in the areas of primary and early secondary prevention of the disease and its complications.

In relation to that, the aim of the present work was to contribute to the clarification of possible relationships among selected markers of inflammation, oxidative damage to nucleic acids, and MetS in the pathogenesis of psoriasis and associated complications. We evaluated (1) the levels of systemic inflammation (characterized by the level of CRP) and oxidative damage to nucleic acids found in both the group of patients and in the group of controls, depending on the presence (or absence) of MetS; and (2) the possible relationships between all measured variables in both of the above mentioned groups.

## 2. Results

Table 1 presents the basic characteristics of the subjects in the experimental group and control group, including the PASI score (Psoriasis Area Severity Index score), the diagnostic criteria of the MetS, the level of CRP, and the level of DNA/RNA damage. In the group of patients, the MetS diagnosis criteria were met by 23 subjects (62%), and in the group of controls, 18 subjects met these criteria (42%). The difference in the presence of MetS in patients and controls was not significant. Significantly elevated levels of fasting glucose, TAG (triglycerides), and high-density lipoprotein (HDL cholesterol) were found in the group of patients (*p* < 0.01; *p* < 0.05; *p* < 0.001). Similarly, the group of patients showed significantly elevated levels of CRP (*p* < 0.001) and DNA/RNA damage (*p* < 0.001), when compared with the controls.

**Figure 1:**
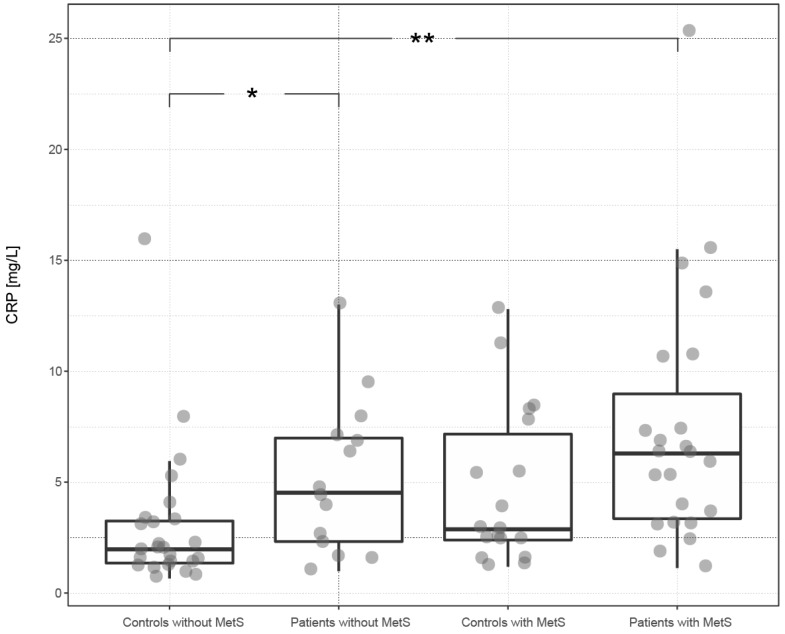
The distribution of CRP (C-reactive protein) among subgroups of subjects defined by the presence of psoriasis and metabolic syndrome. The box plot illustrates the distribution of CRP among subgroups of subjects defined by the presence of psoriasis and MetS (metabolic syndrome). Significant differences are depicted in the upper part of the plot, where * corresponds to *p* < 0.05 and ** to *p* < 0.01. For details, see Table 2. In the plot, the lower and upper hinges (first and third quartiles) surround the median of subgroup CRP distribution. The whiskers extend to the largest or smallest value no further than 1.5 times the distance between the first and third quartiles.

**Figure 2:**
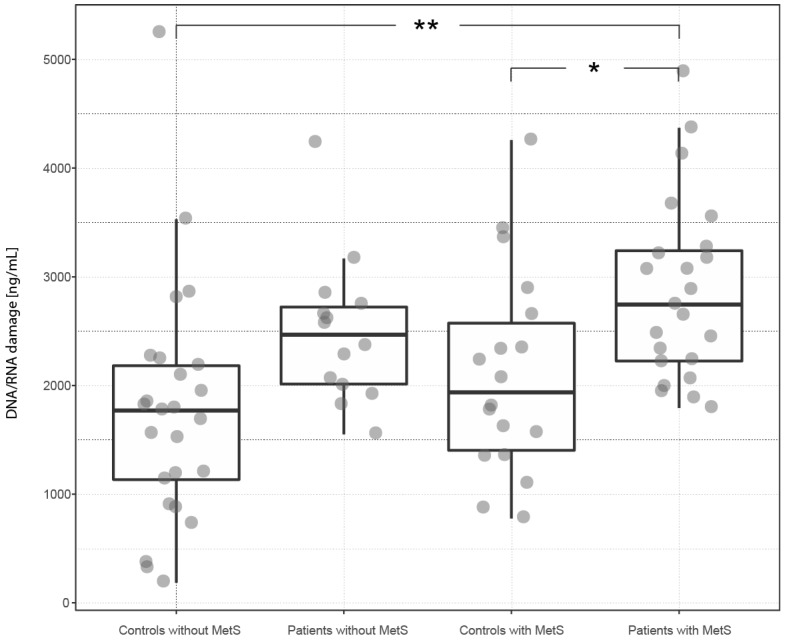
The distribution of DNA/RNA damage among subgroups of subjects defined by the presence of psoriasis and MetS. The box plot illustrates the distribution of DNA/RNA damage among subgroups of subjects defined by the presence of psoriasis and MetS. The plot arrangement corresponds to Figure 1.

Table 2 and Figure 1 and Figure 2 present levels of systemic inflammation (expressed as CRP) and DNA/RNA damage in the groups of patients and controls, depending on the presence (or absence) of MetS. Psoriasis increased the level of CRP by 117% in the subgroup of patients with MetS and by 125% in the subgroup of patients without MetS (compared to the corresponding controls with MetS and without MetS). The presence of MetS increased the level of CRP by 40% in patients and by 45% in controls (versus patients and controls without MetS). The highest level of CRP was found in the subgroup of patients with MetS and the lowest in the subgroup of controls without MetS. Statistically significant differences were found between the subgroup of patients with MetS and the subgroup of controls without MetS (*p* < 0.01) and between the subgroup of patients without MetS and the subgroup of controls without MetS (*p* < 0.05).

Psoriasis increased the level of DNA/RNA damage by 42% in the subgroup of patients with MetS and by 39% in the subgroup of patients without MetS (compared to the corresponding controls with MetS and without MetS). The presence of MetS increased the level of DNA/RNA damage by 11% in patients and by 9% in controls (versus patients and controls without MetS). The highest level of DNA/RNA damage was found in the subgroup of patients with MetS and the lowest in the subgroup of controls without MetS. Statistically significant differences were found between the subgroup of patients with MetS and the subgroup of controls without MetS (*p* < 0.01), and between the subgroup of patients with MetS and the subgroup of controls with MetS (*p* < 0.05).

**Figure 3:**
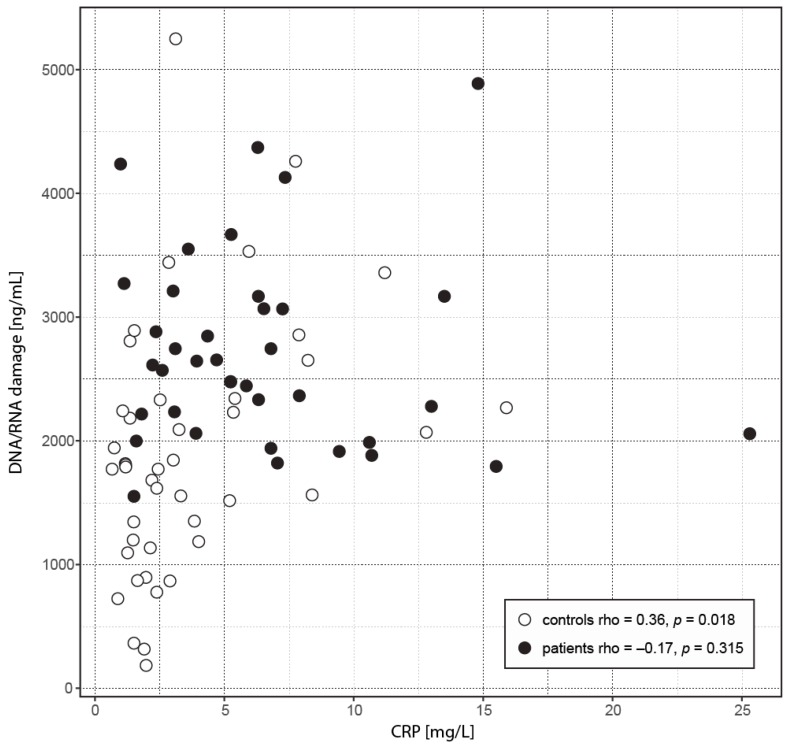
The relationship between CRP and DNA/RNA damage. The scatterplot depicts a relationship between CRP and DNA/RNA damage. Data of patients shows a significant shift toward higher values compared to controls (see Figure 1 and Figure 2) and also a different relationship (Fisher *p* = 0.0237). While in controls the correlation is positive, the relationship found in patients was not significantly different from zero.

In the attempt to evaluate relationships between inflammation (CRP), DNA/RNA damage, and MetS, we performed correlation analyses. The results identified significant correlations between the PASI score and fasting glucose (rho = 0.38, *p* = 0.021). In exploring other parameters, we assessed, beside the relationship significance, whether the relationships are different between the groups. This was true for a positive correlation between DNA/RNA damage and CRP (rho = 0.36, *p* = 0.018) in the controls, which significantly differed (Fisher *p* = 0.0237) from the relationship found in the patients (rho = −0.17, *p* = 0.315) (Figure 3). We did not find such significant between-group difference for other significant correlations: age vs. CRP in patients (rho = 0.39, *p* = 0.016) and controls (rho = −0.024, *p* = 0.877)—Fisher *p* = 0.067; waist circumference vs. CRP in patients (rho = 0.36, *p* = 0.028) and controls (rho = 0.04, *p* = 0.821)—Fisher *p* = 0.157. 

To uncover the roles of monitored parameters in disease severity, we modeled the PASI score as a linear combination of predictors: DNA/RNA damage, CRP, waist circumference, fasting glucose, HDL cholesterol, triglycerides, and systolic and diastolic blood pressure. The regression analysis showed that such a model did not sufficiently explain PASI variability (adjusted *R*^2^ = 0.16, *p* = 0.128), and only after the optimization and reduction of nonsignificant parameters pointed to a model with CRP, DNA/RNA damage, diastolic blood pressure, and glucose (adjusted *R*^2^ = 0.24, *p* = 0.014). The diastolic blood pressure (*p* = 0.015) and glucose (*p* = 0.040) contributed the most to the explained variance. An even better model of the PASI score might be achieved using selected parameters with second order interactions (*R*^2^ = 0.53, *p* < 0.001). It was not surprising that this model with 10 predictors described 36 observations well; however, validation of this model did not converge to such a good fit in split groups (first 18 patients: *R*^2^ = 0.61, *p* = 0.008 vs. last 18 patients: *R*^2^ = 0.26, *p* = 0.238).

## 3. Discussion

The concept of psoriatic pathophysiology can be proposed as a cascade of events starting from inflammation of the skin, predisposing to systemic inflammation, increased oxidative stress, and ultimately leading to many comorbidities, such as MetS [20].

Literary sources describe the high probability of co-occurrence of psoriasis and MetS [4,19]. A higher prevalence of MetS in psoriatic patients has been found compared with controls (62% vs. 42%) in our study (Table 1). This is in congruity with a number of studies from diverse geographic locations [19].

However, it must be mentioned that some other research papers have shown no significant difference in the prevalence of MetS between psoriasis patients and controls. These results may differ because of various criteria applied in assessing MetS (different genetic and epigenetic predispositions such as particular lifestyles) [19,21].

The relationship between psoriasis and the MetS has not been completely clarified. However, it was described that both entities have a common pathophysiology, specifically underlying chronic inflammation [19,22]. Some studies indicate that the severity of psoriasis is associated with MetS (and related cardio-metabolic risk factors) in a “dose-response” manner, from mild to severe psoriasis [19,23]. On the other hand, some other studies have not demonstrated this association. Such a finding suggests that psoriatic patients with a different intensity of disease might share a similar possibility to develop the cardio-metabolic risk factors [19,24].

In our study, the group of patients had an insignificantly higher level of abdominal obesity (waist circumference). A statistically significant difference (compared to controls) was not achieved even after the division of the groups into subgroups of men and women (Table 1). The finding of higher levels of abdominal obesity in psoriatic patients is consistent with the results of other authors [3,19,25]. The question remains whether abdominal obesity contributes to the formation of psoriasis, or whether it is a natural consequence of psoriasis. It has been shown that lifestyle factors and chronic inflammation are associated with psoriasis and promote obesity. On the other hand, obesity as a chronic inflammatory condition is associated with elevated levels of different inflammatory factors that may contribute to the development of psoriasis [19,25].

Similarly to other articles, we found significantly elevated levels of fasting glucose (*p* < 0.01) in the group of psoriatic patients (Table 1) [21]. In their recent review work, Armstrong et al. reported that psoriasis is associated with an increased prevalence of diabetes mellitus (elevated levels of fasting glucose) and increased risk of developing diabetes mellitus [21]. Psoriasis and diabetes mellitus likely have a pathophysiologic link and there is evidence that insulin resistance can influence the pathogenesis of MetS [19].

Although most studies indicate hypertension as a frequent component of MetS in psoriatic patients, in our study we did not find a significantly higher prevalence of hypertension in the group of patients (Table 1) [21]. It should be noted that a similar phenomenon was also observed in our previous work with another group of psoriatic patients [4]. We believe that this finding could be related to the heterogeneity of the severity of the disease in the group of patients. Some studies indicate that although the level of cardiovascular risk factors (including hypertension) was higher in severe psoriasis, no association between psoriasis severity and risk of hypertension was found [19,26]. It must be admitted that the exact mechanism underlying the association between psoriasis and hypertension is still unknown.

We found significantly elevated levels of TAG and HDL cholesterol (Table 1) in the group of patients (*p* < 0.05 and *p* < 0.001). It is known that both psoriasis and MetS are associated with abnormal lipid metabolism [27]. Some authors proposed that some form of relation between psoriasis and atherogenic dyslipidemia exists, including increased levels of TAG, total cholesterol, low-density lipoproteins (LDL cholesterol), and very low-density lipoproteins (VLDL cholesterol) and a lower level of HDL cholesterol in patients [28].

However, it must be noted that there are studies contraindicating the above mentioned relation. The discrepancy goes so far that some studies indicate a normal or even lower serum TAG level and normal serum level of HDL cholesterol in psoriatic patients [27,29]. It is a little difficult to interpret our finding of a higher serum level of HDL cholesterol in the group of patients (*p* < 0.001). Although following the division of the groups of patients and controls into subgroups of men and women led to a reduction of the significance of the differences (men non-significant; women *p* < 0.01), the HDL cholesterol levels were still higher in subgroups of patients. We believe that this unusual finding could be (to some extent) attributed to gender heterogeneity in the study design. Specifically, the patient group was composed of 22 women (59%) and 15 men (41%), while the control group was composed of 19 women (44%) and 24 men (56%). Moreover, some authors stated that abnormalities in lipid metabolism in psoriatic patients may be genetically determined [19,30].

Some authors described elevated levels of CRP in psoriatic patients with MetS in comparison to patients without MetS [5,6]. The group of our psoriatic patients had a significantly elevated level of CRP, compared to the control group, regardless of the presence or absence of MetS. Subgroups of persons (patients and controls) with MetS had insignificantly increased levels of CRP, compared to those without MetS (Table 1 and Table 2; Figure 1).

The group of psoriatic patients had a significantly elevated level of DNA/RNA damage, compared to the control group, regardless of the presence or absence of MetS. Subgroups of persons (patients and controls) with MetS had insignificantly increased levels of DNA/RNA damage, compared to those without MetS (Table 1 and Table 2; Figure 2).

Oxidative stress (DNA/RNA damage) is one of the central pathologic factors in psoriasis [31]. Some studies have revealed significantly increased levels of oxidative stress markers in psoriatic patients, including plasma or serum [14,31,32]. In our previous study, we found that oxidative stress in patients with psoriasis is further increased by certain types of treatment, for example, Goeckerman therapy [14]. The used method of measurement relies upon the macromolecule of present interest, e.g., nucleic acids. DNA integrity is protected from oxidative damage by a multitude of effective repairing mechanisms, such as base excision repair and nucleotide excision, dedicated to coping with oxidative damage to DNA. To prevent DNA damage, oxidized nucleobases are excised and eliminated [11].

To express the intensity of oxidative stress, we used (in our study) the combination of 8-hydroxy-2′-deoxyguanosine released from DNA, 8-hydroxyguanosine from RNA, and 8-hydroxyguanine from either DNA or RNA. The 8-hydroxyguanosine is the oxidative modification of the guanosine nitrogen base in DNA, and acts as the most extensively studied marker of oxidative stress and DNA damage in humans. The serum level of the 8-hydroxy guanosine level could be a potential non-specific biomarker for the early diagnosis of psoriasis and its management [14,15,32].

To estimate the possible importance of the monitored parameters on disease severity, we used systemic inflammation (CRP), oxidative damage to nucleic acids, and factors reflecting MetS as predictors of the PASI score in a general linear model. The model showed that the aforementioned parameters are not good predictors and explained only 35% (16% adjusted for number of predictors) of the PASI variability. By optimizing the model by removing parameters not contributing to the PASI description we achieved up to 24 % of the adjusted and explained PASI variability and the model gained statistical significance (*p* = 0.014). Parameters with the highest predictive power in our model were the fasting glucose and the diastolic blood pressure, both markers of the MetS.

In addition to the aforementioned differences of absolute values between the control group and the patients, our correlation analysis showed a different relationship between the monitored parameters. While the link between the systemic inflammation (CRP) and oxidative damage to nucleic acids was positive in the control group, this relationship was disrupted and significantly different in the patient group. Correlation analysis does not reflect causality, but it demonstrates a specific regulatory process of the systemic inflammatory reaction (CRP) and oxidative damage to nucleic acids in psoriasis. Our results show that the increase in DNA/RNA damage is caused (in given study) by psoriasis and MetS. Unlike in our previous work [14], we can exclude the influence of therapy. The patients with psoriasis in the present study did not have any form of treatment three months before the study.

The questions of whether psoriasis is a predilection for MetS or whether metabolic disorders can predispose patients to psoriasis remain unanswered. In agreement with the authors Milcic et al. [19], we suppose that the higher prevalence of MetS in patients with psoriasis indicates the urgency for the early detection of metabolic disorders and proper treatment of psoriasis.

Finally, several limitations of the present study should be mentioned. Firstly, when generalizing the study conclusions, we must take into account (a) the fact that the sets of persons are relatively small, and (b) the possibility of mistakes associated with the non-random selection of patients from one clinical center (selection and information bias). Secondly, the results could have been affected by the gender imbalances in the monitored groups.

## 4. Materials and Methods

### 4.1. Study Groups

The experimental group consisted of 37 patients with psoriasis (22 women and 15 men; median age: 58 years; age range: 25–68 years). The patients were investigated at the Clinic of Dermal and Venereal Disease, Charles University Hospital in Hradec Kralove. 

The control group included 43 healthy blood donors (19 women and 24 men; median age: 56 years; age range: 45–59 years) from the Department of Transfusion Medicine, Charles University Hospital in Hradec Kralove. For the study purposes, all subjects in both groups were divided into subgroups depending on the presence of MetS (patients with MetS, patients without MetS, controls with MetS, and controls without MetS).

All subjects gave their informed consent for inclusion before they participated in the study. The persons with inflammatory diseases (such as infectious diseases, malignancy, and inflammatory rheumatic diseases), pregnancy, and those using nonsteroidal or anti-inflammatory medications were excluded from the study. Patients with psoriasis did not have any form of psoriasis treatment three months before the study.

The study was conducted in accordance with the Declaration of Helsinki, and the protocol was approved by the Ethics Committee of the Charles University Hospital in Hradec Kralove, Czech Republic (Project identification code PROGRES Q40-09 and Q40-10, reference number 201705 I83P, date 2 May 2017).

### 4.2. Systemic Inflammation (CRP)

The level of systemic inflammation was assessed by the level of CRP. Peripheral blood samples were collected from the cubital vein of all persons in both groups (by BD Vacutainer sampling tubes; BD Biosciences, San Jose, CA, USA). Blood serum samples were isolated by centrifugation and stored at −70 °C until analysis. Repeated thawing and freezing was avoided. The level of CRP was determined by immuno-nephelometry on IMMAGE 800 (Beckman, Brea, CA, USA) and results were expressed in milligrams (mg) per liter of serum with a detection limit of 1.0 mg per liter.

### 4.3. Oxidative Damage to Nucleic Acids

Peripheral blood samples were collected from the cubital vein of all persons in both groups (by BD Vacutainer sampling tubes). Blood serum samples were isolated by centrifugation and stored at −70 °C until analysis. Repeated thawing and freezing was avoided. The level of oxidative damage to nucleic acids (DNA/RNA damage) was determined by an EIA Kit (Enzyme Immunoassay, Cayman Chemical Company, Ann Arbor, MI, USA). The damage was expressed as the sum of three oxidized guanine species in serum: 8-hydroxy-2′-deoxyguanosine released from DNA, 8-hydroxyguanosine from RNA, and 8-hydroxyguanine from either DNA or RNA. The level of DNA/RNA damage was calculated in picograms (pg) of all guanine species per milliliter of serum, with a detection limit of 33 pg of species per milliliter of serum.

### 4.4. Metabolic Syndrome

Evaluation of the presence of MetS in observed subjects was done in accordance with the criteria of the National Cholesterol Education Program Adult Treatment Panel (NCE/ATPIII) [33]. The diagnosis of MetS can be declared when three of the five listed criteria are present: (1) increased waist circumference or abdominal obesity (≥102 cm for men and ≥88 cm for women); (2) glucose intolerance presented by higher fasting glucose ≥5.6 mmol/L or known treatment for diabetes; (3) raised level of triglycerides (TAG) ≥1.7 mmol/L; (4) reduced level of high-density lipoproteins (HDL cholesterol) <1.03 mmol/L for men and <1.30 mmo/L for women; and (5) elevated blood pressure (systolic blood pressure ≥130 mmHg, and/or diastolic blood pressure ≥85 mmHg). The waist circumference (abdominal obesity) and the systolic and diastolic blood pressure were measured at the Clinic of Dermal and Venereal Diseases (patients) and at the Department of Transfusion Medicine (controls) (both at Charles University Hospital and Faculty in Hradec Kralove). The same experienced staff performed measurements in both groups. The levels of fasting glucose, TAG, and HDL cholesterol in peripheral blood samples (from the cubital vein) were analyzed in the serum by standard laboratory metods at the Institute of Clinical Biochemistry and Diagnostics (Charles University Hospital and Faculty in Hradec Kralove).

### 4.5. Disease Status Determination

The condition of the disease was assessed using a clinical evaluation of Erythema, Desquamation, and Skin infiltration (Psoriasis Area Severity Index; PASI score) [34].

### 4.6. Statistical Analysis

The data were statistically processed by the R software version 3.4 (Available online: http://www.R-project.org/) [35] using the “nortest” package [36]. When the Anderson-Darling test for normality had rejected the hypothesis of a normal distribution of data, a nonparametric two-side Wilcoxon sum rank test was used; otherwise, the *t*-test was employed for intergroup comparisons. Relationships among outcome measurements were evaluated using a Pearson or Spearman correlation test, depending on data normality. A between group correlations comparison was based on z-score comparisons [37]. The role of oxidative stress in nucleic acids and metabolic markers (waist circumference, systolic and diastolic blood pressure, fasting glucose, TAG, and HDL cholesterol) on CRP or PASI was assessed using multiple linear regression analysis. Based on normality and Bartlett’s test of variances, ANOVA or the Kruskal-Wallis Rank Sum Test were used in the case of two factor comparisons, the patient vs. control group, and the presence of metabolic syndrome. For significant differences, we performed pairwise Student’s *t*-tests or Wilcoxon rank sum tests, appropriately. We considered differences as statistically significant when the probability level (*p*) was below 0.05, appropriately corrected for multiple comparisons.

## 5. Conclusions

Our findings demonstrate that both psoriasis and MetS individually contribute to the increase in systemic inflammation and oxidative damage to nucleic acids. The psoriasis contributes to an increase in the levels of both effects more significantly than MetS.

The correlation analysis showed an impaired relationship of the markers of systemic inflammation and oxidative damage to nucleic acids in psoriasis. The regression analysis pointed out that metabolic syndrome, namely the fasting glucose level and diastolic pressure, are closely related to psoriasis severity (PASI).

## Figures and Tables

**Table 1:** The basic characteristics of the subjects in the experimental (patients) and control group, the PASI score (Psoriasis Area Severity Index score), the diagnostic criteria of the MetS (metabolic syndrome), the level of CRP (C-reactive protein), and the level of DNA/RNA damage.

Variable	Patients (*n* = 37)	Controls (*n* = 43)	*p*-Value
Age	58 (53–62)	56 (52–60)	0.230 ^A^
Gender (men/women)	15/22	24/19	-
PASI score	15.8 (12.0–20.8)	-	-
With/without MetS	23/14	18/25	0.078 ^C^
Waist circumference (cm)	97 (86–108)	96 (87–102)	0.200 ^B^
Waist circumference men (cm)	101 (90–119)	100 (90–104)	0.366 ^B^
Waist circumference women (cm)	97 (87–102)	87 (84–99)	0.161 ^B^
Fasting glucose (mmol/L)	5.7 (5.3–6.9)	5.1 (4.7–6.3)	0.007 ^A,^**
TAG (mmol/L)	1.6 (1.1–2.1)	1.1 (0.8–1.7)	0.040 ^A,^*
HDL cholesterol (mmol/L)	1.2 (1.1–1.5)	1.0 (0.9–1.2)	<0.001 ^A,^***
HDL cholesterol, men (mmol/L)	1.1 (1.0–1.4)	1.0 (0.9–1.1)	0.079 ^A^
HDL cholesterol, women mmol/L)	1.3 (1.2–1.6)	1.2 (1.0–1.2)	0.002 ^B,^**
Systolic blood pressure (mmHg)	135 (130–150)	140 (128–150)	0.463 ^B^
Diastolic blood pressure (mmHg)	90 (80–90)	85 (80–90)	0.210 ^A^
CRP (mg/L)	5.9 (3.1–7.4)	2.4 (1.5–4.6)	<0.001 ^A,^***
DNA/RNA damage (pg/mL)	2612 (2059–3168)	1788 (1191–2298)	<0.001 ^A,^***

All data are presented as medians and lower and upper quartiles in brackets; For abbreviations see the chapter Material and Methods; ^A^ Wilcoxon rank sum test, ^B^ Student *t*-test, ^C^ Fisher’s Exact Test for Count Data; Statistical significance: * *p* < 0.05; ** *p* < 0.01; *** *p* < 0.001.

**Table 2:** The level of oxidative damage to nucleic acids and the level of systemic inflammation (CRP) in the group of patients and in the group of controls, depending on the presence of MetS (metabolic syndrome).

**Variable**	**Patients with MetS** **(*n* = 23)**	**Controls with MetS** **(*n* = 18)**	***p*-Value**
CRP (mg/L)	6.3 (3.4–9.0)	2.9 (2.4–7.2)	0.108 ^A^
DNA/RNA damage (pg/mL)	2745 (2225–3241)	1937 (1403–2573)	0.049 *
	**Patients without MetS** **(*n* = 14)**	**Controls without MetS** **(*n* = 25)**	
CRP (mg/L)	4.5 (2.3–7.0)	2.0 (1.4–3.3)	0.049 ^A,^*
DNA/RNA damage (pg/mL)	2467 (2013–2722)	1770 (1134–2182)	0.086
	**Patients with MetS** **(*n* = 23)**	**Patients without MetS** **(*n* = 14)**	
CRP (mg/L)	6.3 (3.4–9.0)	4.5 (2.3–7.0)	0.311 ^A^
DNA/RNA damage (pg/mL)	2745 (2225–3241)	2467 (2013–2722)	0.677
	**Controls with MetS** **(*n* = 18)**	**Controls without MetS** **(*n* = 25)**	
CRP (mg/L)	2.9 (2.4–7.2)	2.0 (1.4–3.3)	0.077 ^A^
DNA/RNA damage (pg/mL)	1937 (1403–2573)	1770 (1134–2182)	0.677

All data are presented as medians and lower and upper quartiles in brackets; For abbreviations see the chapter Material and Methods; ^A^ Kruskal-Wallis rank sum test pro significant result followed by Wilcoxon signed rank tests; Statistical significance: * *p* < 0.05; ** *p* < 0.01.
